# Dietary intakes and nutritional adequacy of Australians consuming plant-based diets compared to a regular meat-eating diet

**DOI:** 10.1038/s41430-025-01621-1

**Published:** 2025-04-18

**Authors:** Grace Austin, Jessica J. A. Ferguson, Shaun Eslick, Christopher Oldmeadow, Lisa G. Wood, Manohar L. Garg

**Affiliations:** 1 School of Biomedical Sciences & Pharmacy, University of Newcastle, Callaghan, NSW Australia; 2 Food and Nutrition Research Program, Hunter Medical Research Institute, New Lambton Heights, NSW Australia; 3 Macquarie Medical School, Macquarie University, Macquarie Park, NSW Australia; 4 School of Health Sciences, University of Newcastle, Callaghan, NSW Australia; 5 Clinical Research Design, Information Technology and Statistical Support Unit, Hunter Medical Research Institute, New Lambton, NSW Australia

**Keywords:** Biomarkers, Risk factors

## Abstract

**Background/Objectives:**

Despite the healthful nature of plant-based diets (PBDs) there is potential for nutritional inadequacies. This study aimed to compare dietary intakes and nutritional adequacy in Australians following PBDs compared a regular meat-eating diet.

**Subjects/methods:**

A cross-sectional study of adults (*n* = 240) aged 30–75 yrs, habitually following dietary patterns for ≥6 months; vegan, lacto-vegetarian, pesco-vegetarian, semi-vegetarian or regular meat-eater (*n* = 48 per group). Dietary intakes were assessed using validated food frequency questionnaires and dietitian-administered diet histories. Multivariable regression adjusted for sex, age, BMI, race, energy intake and physical activity.

**Results:**

Compared to regular meat-eaters, vegans and lacto-ovo vegetarians had significantly lower intakes of protein (4-5 EN%), saturated fat (2-4 EN%), trans fat, cholesterol, vitamin B_12_, iodine, riboflavin, niacin, sodium, and long-chain omega-3 polyunsaturated fatty acids (LCn-3PUFA), and higher carbohydrate (5-7 EN%), fibre, vitamin E, folate, magnesium, iron, and n-6PUFA, whereas, pesco-vegetarians and semi-vegetarians had intermediate intakes. Individuals following PBDs consumed significantly more daily serves of vegetables (1–1.5 serves), fruit (1 serve, vegan only), legumes/nuts (0.5–1 serves), and less discretionary choices (0.5–1 serves) compared to regular meat-eaters. All dietary patterns were adequate in protein, exceeded in fat, inadequate in carbohydrate and met recommended serves of fruit and vegetables, but not grains. Inadequate serves were observed for meat/poultry/eggs/beans/nuts among PBDs except pesco-vegetarians, and dairy among semi-vegetarians and regular meat-eaters. Vegans and lacto-vegetarians were inadequate in vitamin B12, LCn-3PUFA, iodine, and in addition calcium among vegans, iodine in pesco-vegetarians, and LCn-3PUFA in semi-vegetarians and regular meat-eaters.

**Conclusion:**

PBDs, while higher in beneficial nutrients and wholefood groups than regular meat-eaters, may lead to nutritional inadequacies if not planned appropriately.

## Introduction

The adoption of plant-based diets (PBDs) is becoming increasingly popular worldwide [1, 2]. Influences that underpin the adoption of PBDs include the overall positive perception by the public, environmental sustainability, animal welfare, ethics [3, 4] and the potential health benefits such as weight loss effects [5, 6] and reduced risk of type two diabetes (T2D) [7]. Literature surrounding PBDs use different definitions to distinguish dietary patterns, however they are generally characterised by reduced intakes of animal meats, dairy and eggs and higher intakes of plant-based foods including vegetables, grains, legumes, nuts and fruit [8].

Revised dietary guidelines across the globe have emphasised the inclusion and/or adoption of more plant-based foods and PBDs in accordance with the growing scientific evidence for their potential health benefits and emerging recognition of environmental impact [9–12]. The latest American Dietary Guidelines (2020–2025) highlights the benefits of PBDs such as vegetarian-style and Mediterranean-style as variations that exemplify a healthy dietary pattern [9]. Furthermore, the Danish Official Dietary Guidelines (2021) [11] and Canada’s Food Guide (2019) [10] refer to plant-based proteins and recommend plant-based foods as preferable sources over animal-based proteins. In contrast, the current Australian Dietary Guidelines (2013), with greater emphasis on the inclusion of animal foods such as meats and dairy, make no specific recognition towards prioritising plant-forward dietary patterns [13]. These guidelines are currently under review and the examination of animal versus plant sources of protein was identified as a priority research area by the National Health and Medical Research Council [14]. Therefore, it is even more poignant that the dietary intake and adequacy of Australians habitually following various PBDs is better understood.

Assessing the nutritional adequacy of individuals consuming various PBDs is essential to evaluate potential risk of chronic disease development. Vegan and vegetarian dietary patterns have shown to be associated with lower risk of cardiovascular disease (CVD) [15, 16], metabolic syndrome [17], T2D [18], obesity [5, 6], and some cancers [19]. Additionally, they may be more environmentally sustainable than diets rich in animal products [20]. Reported health benefits of PBDs may be linked to their more favourable nutrient composition comprised of lower intakes of saturated fat and cholesterol and higher intakes of dietary fibre [21, 22]. On the other hand, compared to dietary patterns that includes animal-based food products those following PBDs may be at risk of nutritional inadequacy including protein, vitamin B_12_, long chain omega-3 polyunsaturated fatty acids (LCn-3PUFA), vitamin B_2_, vitamin D, calcium, zinc [23], iron, and iodine [24].

Current understanding of the dietary intake of individuals following various PBDs stems from secondary analyses of observational studies conducted overseas [23, 25]. Previous studies have not cohesively defined PBDs, warranting a more collective categorising methodology to appropriately compare and distinguish differences between different PBDs, as well as draw conclusions on their health impact. The current study is the only Australian-based study reporting dietary intake from individuals who were purposefully sampled as habitually consuming various PBDs as part of the primary study design. Moreover, 80% of the study sample reported to follow various PBDs [26] and the rationale has been previously published [26]. To help create universal understanding of the definitions associated with various PBDs, this study used definitions previously implemented by Australian-based cohorts [7, 27, 28] originally adapted from Mihrshahi et al. [29] and aligned with the World Health Organisation [30].

The primary aim is to investigate the dietary intake and nutritional adequacy of individuals following various plant-based compared a regular meat-eating diet characterised by inclusion or exclusion or animal meats, dairy, and eggs, in accordance with national dietary guidelines. This novel, first-time, Australian, population-based study specifically recruiting a sample following PBDs is paramount to help inform national nutrition policy and population-based dietary guidelines that are emerging with a prioritisation towards more plant-based dietary characteristics.

## Methods

### Participants and study design

The protocol for this cross-sectional study has been described in detail elsewhere [26]. Participants attended one data collection timepoint between November 2021 and March 2023 at the Nutraceuticals Research Program, School of Biomedical Sciences & Pharmacy, University of Newcastle, Callaghan NSW, Australia. Data were collected from consented enrolled participants after an overnight fast (~10–12 h) by the lead investigator. Participants were deemed eligible if they were adults aged 30–75 years and following a dietary pattern for ≥6 months. They were excluded if they had current or previous diagnosis of CVD, were pregnant or breast feeding or made any significant changes to their usual food intake or physical activity regime in the past 6 months. An eligibility screening criteria published elsewhere [26] was used to assessed weekly consumption of meat, seafood, eggs, and dairy to categorise participants into dietary pattern groups. Individuals who were habitually consuming one of five dietary patterns (48=per group) were recruited into the following groups: vegan (nil animal-based foods), lacto-ovo vegetarian (LOV, nil meat, ± eggs, ± dairy), pesco-vegetarian (PV, nil meat, seafood consumption ≥1 per week, ± dairy, ± eggs), semi-vegetarian (SV, meat consumption ≤2 per week) or RME (meat consumption ≥7 per week). This study has been approved by the University of Newcastle’s Human Research Ethics Committee (HREC 2020-0195). This trial has been registered with the Australian New Zealand Clinical Trials Registry (ACTRN12621000743864).

### Participant characteristics

Questionnaires included a self-reported medical and demographic histories which collected variables such as age, race, sex, ethnicity, smoking status, level of education, marital status, prescribed or over-the-counter medication(s) and habitual supplement use. The International Physical Activity Questionnaire (IPAQ, Long Version October 2002) is a self-administered and validated questionnaire which was used to assess habitual physical activity levels interpreted as metabolic equivalent of task minutes per week (MET/week) to measure the level of energy required for physical activities [31]. Overweight and obese classifications were based on a BMI of ≥25 kg/m^2^ or ≥30 kg/m^2^ respectively [32]. Lead investigator collected a seated blood pressure (three serial measurements using an average of the final two).

### Assessment of dietary intake

The Australian Eating Food Survey (AES^®^) food frequency questionnaire (FFQ) was used to measure qualitative intake of food groups per day. The AES^®^ has been validated in Australian populations and is an online self-administered 120-question FFQ which examines food and nutrient intake over the preceding 3–6 months [33, 34]. The assessment of habitual intake of food items relating to a food category over a longer period of time is most accurately determined using FFQs [35]. The AES^®^ FFQ offers 6–8 options for the daily, weekly, or monthly intake of foods ranging from ‘Never’, ‘less than 1 per month’, ‘1–3 per month’, ‘1 per week’, ‘2–6 per week’, ‘1 per day’, ‘2–3 per day’ and ‘4 or more per day’. Frequency of foods consumed were converted to daily equivalents and the total reported intakes of all questions related to a specific food group was recorded. There were 19 questions related to vegetables, 11 related to fruit, 22 related to grains, 29 related to ‘protein-rich foods’ (meats, poultry, seafood, eggs, legumes and nuts), 9 related to animal-based dairy’ and 26 related to discretionary foods (including sugar sweetened beverages). Daily intake of food groups and food categories were compared to the Australian Guide to Healthy Eating (AGTHE) recommendations. Recomended daily food group serves used to calculate adequacy were based on the appropriate age and sex recommendations for each participant. There is currently no available validated FFQ which evaluates consumption of various plant-based meat alternatives (PBMAs) and plant-based dairy alternatives (PBDAs). In the absence of this, an Accredited Practicing Dietitian (APD) administered an in-person FFQ which followed the same closed question format used in the AES FFQ^®^ to obtain intake of PBMAs and PBDAs. Participants were asked ‘*How often do you have the following plant-based alternatives*’ for meat, milk, yoghurt, cheese, and ice-cream (5 questions). Dietitian-administered FFQ have been considered more accurate that self-administered FFQs [36]. The Australian Recommended Food Scores (ARFS) were used to investigate diet quality across groups [37].

To collect quantitative dietary data including total energy intake, macronutrients, and micronutrient, an APD conducted a comprehensive diet history. This method collected usual eating habits over the past month, which is a commonly used duration of assessment [38, 39]. The diet history was comprised of two parts. Part one was a 2-day food record which collected information on individual food items, brands, portion sizes and use of condiments, spreads and oils in preparation methods at usual each eating occasion [40]. This is followed by, part two, a ‘cross-check’ to clarify usual intake of foods over the past month including core food groups, discretionary choices, oils/spreads, eating out occasions, fluids, and alcohol consumption to ensure all food items were accounted for, reducing error [40]. Diet histories are a more accurate means to assess quantitative dietary data than FFQs and been proven as a reliable tool by previous research demonstrating similar results to that of a weighed food record [35, 41]. Data were analysed using version 10 ‘FoodWorks’ (Xyris®, Brisbane, Australia, sourced online) which sourced nutritional data of food items from ‘AusBrands’ and ‘AusFoods’ (2019) that derives its nutrient data from Australian Food and Nutrient Database. Food products not already included in this database were manually added using the product’s nutrient information panel, inclusive of fortification. Micronutrient and macronutrient data were presented as mean average consumed (mg/g) per day from the two diet histories. Macronutrient intakes were displayed as a percentage of total energy intake and compared to the respective Acceptable Macronutrient Distribution Ranges (AMDR), and micronutrient intake was evaluated against the Australian Nutrient Reference Values (NRVs), as per the Australian dietary guidelines [42]. NRVs used to calculate nutrient adequacy were based on the appropriate age and sex recommendations for each participant.

### Statistical analyses

Data were assessed for normal distribution via inspection of histograms and quantile plots and continuous data were expressed as means ± SD and categorical data as counts (*n*) and frequencies (%). One-way ANOVA was applied to parametric data, Kruskal-Wallis test to non-parametric data and Fisher’s Exact tests was used assess categorical data. Participant characteristics were compared across the dietary patterns groups to identify potential confounders variables. Variables with the *p*-values of these tests that were less than 0.05, along with those deemed important based expert opinion and literature (sex, age, BMI, race, total energy intake and physical activity) were considered as potential confounding variables. For each outcome, a multivariable regression model was employed, treating dietary patterns as the exposure factor, and incorporating the relevant potential confounding variables as covariates [25]. To assess nutritional adequacy, nutrient/food group intakes were compared to NRVs/AGTHE recommendations specific to the age and sex for each participant. Nutrient intake as a percentage of the NRVs/AGTHE recommendation was adjusted for in the previously described regression analysis. Adequacy was rounded to the nearest 5% for all descriptive results. Adjusted means and % CIs were presented rather than mean differences or multiple comparisons to inspect daily intakes and levels of adequacy relevant across all groups including the control group allowing better translation to clinical practice [25]. Adjusted *P*-values and post hoc comparisons are presented in tables to display differences across groups. Other diagnostic procedures included inspecting residual plots for normality and homogeneity of variance. *P*-values presented are compared to a 5% significance threshold and differences that meet a Bonferroni threshold were also reported. Statistical analyses were conducted using StataCorp. 2016 (Stata Statistical Software: Release 17 (College Station, TX, USA: StataCorp LP)).

## Results

### Participant characteristics

240 participants were recruited into the study evenly across five dietary patterns (48 per group) (Table 1). Participants were aged 54 ± 10 years, more than three quarters of the participants were female (78%), forty-two percent of participants had overweight or obesity with an average BMI of 24 ± 4 kg/m^2^ [43], most (88%) had a higher education and had comparable high levels of physical activity (METs/week) [26]. Age, dietary pattern length and supplement use including iron, vitamin B_12_, and LCn-3PUFA; eicosapentaenoic acids (EPA), and docosahexaenoic acid (DHA) were significantly different across groups. Almost two thirds of participants reported vitamin and mineral supplement use and those following a PBD reported higher use, than the RME diet. Use of iron and vitamin B_12_ supplements were higher in those following a PBD, specifically vegans, compared to RMEs. In contrast, RMEs has the highest use of LCn-3PUFA supplements compared to those adhering to a PBD [26]. Use of other supplements and complementary bioactives including vitamin C, multivitamins, vitamin D, calcium, magnesium, vitamin B complexes, zinc, and curcumin/turmeric were comparable across dietary patterns. Further details on participant characteristics have been published elsewhere [26].

**Table 1:** Participant characteristics across dietary patterns.

	Total sample (*n* = 240)	Vegan (*n* = 48)	Lacto-ovo vegetarian (*n* = 48)	Pesco-vegetarian (*n* = 48)	Semi-vegetarian (*n* = 48)	Regular meat-eater (*n* = 48)	*P*
Females^1^	186 (77.5)	34 (70.0)	36 (75.0)	39 (81.3)	40 (83.3)	37 (77.1)	0.625
Age (yrs)^1^	53.8 ± 10.3	47.8 ± 10.0^a^	53.7 ± 10.0^b^	55.8 ± 11.0^b^	55.2 ± 8.7^b^	56.5 ± 9.7^b^	<0.001
Ethnicity							
Oceanian	110 (45.8)	23 (47.9)	20 (41.7)	21 (43.8)	19 (39.6)	27 (56.3)	0.514
European^2^	66 (27.5)	15 (31.3)	14 (29.2)	10 (20.8)	14 (29.2)	13 (27.1)	0.809
Other^2^	64 (25.7)	10 (20.9)	14 (29.1)	17 (35.4)	15 (31.3)	8 (16.7)	0.238
BMI ≥ 25 kg/m^2 1,3^	100 (41.7)	17 (35.4)	23 (48.0)	20 (41.7)	16 (33.3)	24 (50.0)	0.375
Married	189 (78.8)	40 (83.3)	37 (77.1)	37 (77.1)	35 (73.0)	36 (75.0)	0.511
Physical Activity (MET)^1^	5424 ± 5243	5775 ± 4036	6984 ± 8783	4909 ± 4101	4393 ± 3534	5060 ± 3600	0.134
**Vitamin and Mineral Supplement use (%)**^**4**^							
Supplement users	160 (66.7)	37 (77.1)	36 (75.0)	33 (68.8)	32 (66.7)	22 (45.8)	0.012
Iron	32 9 (13.3)	13 (27.1)	5 (10.4)	6 (12.5)	6 (12.5)	2 (4.2)	0.027
Vitamin B_12_	59 (24.6)	25 (52.1)	9 (18.8)	10 (20.8)	13 (27.1)	2 (4.2)	<0.001
Vitamin C	42 (17.5)	7 (14.6)	13 (27.1)	9 (18.8)	7 (14.6)	6 (12.5)	0.382
Multivitamin	27 (11.3)	6 (12.5)	7 (14.6)	4 (8.3)	7 (14.6)	3 (6.3)	0.605
Vitamin D^1^	73 (30.4)	15 (31.3)	17 (35.4)	19 (39.6)	13 (27.1)	9 (18.8)	0.206
Calcium	35 (14.6)	7 (14.6)	8 (16.7)	7 (14.6)	7 (14.6)	6 (12.5)	0.999
Magnesium	57 (23.8)	11 (22.9)	13 (27.1)	12 (25)	10 (20.8)	11 (22.9)	0.978
Vitamin B complex	25 (10.4)	6 (12.5)	4 (8.3)	9 (18.8)	4 (8.3)	2 (4.2)	0.200
Zinc	26 (10.8)	8 (16.7)	3 (6.3)	6 (12.5)	3 (6.3)	6 (12.5)	0.423
**Complementary Bioactives use (%)**							
Omega-3^1,5^	27 (11.3)	3 (6.3)	7 (14.5)	4 (8.3)	2 (4.2)	11 (22.9)	0.035
EPA/DHA	24 (10.0)	2 (4.2)	5 (10.4)	4 (8.3)	2 (4.2)	11 (22.9)	0.025
ALA	3 (1.3)	1 (2.1)	2 (4.2)	0	0	0	0.514
Curcumin/turmeric	14 (5.8)	4 (9.3)	1 (2.1)	3 (6.3)	3 (6.3)	3 (6.3)	0.801

Data reported as means ± SD for continuous variables and counts and (percentages) for categorical variables. Contentious data was compared using AVOVA and categorical data was compared using Fisher’s Exact.

*ALA* alpha-linolenic acid, *BMI* body-mass index, *EPA* eicosapentaenoic acid, *DHA* docosahexaenoic acid, *MET* metabolic equivalent of task minutes, *WC* waist circumference.

^1^This data has been published elsewhere [28].

^2^European includes North-West & South-East descents. Other races include mixed heritage, North African and Middle Eastern, Peoples of Americas, Australian Aboriginal and/or Torres Strait Islander.

^3^Overweight defined as overweight; BMI ≥ 25 kg/m^2^ and obese; ≥30 kg/m^2^ defined by the WHO [32]. ^4^Participants currently taking medication/supplement as per medical history questionnaire. Supplements consumed ≥3 times per week were reported. Supplements which included ≤3 vitamin/minerals were reported separately and >3 were reported as a multivitamin.

^5^Omega-3 supplements were defined as; EPA/DHA (fish and krill based), and ALA (algae and flaxseed based).

^a,b,c^Values within the same row without a common superscript letter are significantly different (*P* < 0.05).

### Food group intake

After adjustments and compared to RMEs, those following a PBD consumed significantly more daily serves of vegetables (including non-starchy), fruit (vegans only), legumes/nuts, PBDAs, less discretionary choices, and sugar sweetened beverages, had a comparable intake of grains, however, had a lower intake protein-rich foods (Table 2). Compared to RMEs, vegans had a higher intake of fruit by 1 serves/day, and non-starchy vegetable by 1.5 serves/day. Non-meat-eating groups (vegan and LOV) had a lower intake of ‘protein-rich foods’ which was almost half the intake of RMEs (1.3-1.5 Vs 2.7 serves). Those adhering to a PBD consumed two to three times more legumes/nuts compared to RMEs (1.1-1.5 Vs 0.6 serves). Consumption of discretionary choices (including sugar sweetened beverages) were higher among those adhering to a RME diet compared to PBDs by 0.5–1 serves/day. PBDs had a higher ARFS compared to RMEs. Supplementary Table 1 displays unadjusted means of all nutrient intakes and food groups across dietary patterns.

**Table 2:** Adjusted means for quantitative and qualitative dietary intake of macronutrients, micronutrients, and food groups across dietary patterns.

Nutrients/Food groups	Total sample (*n* = 240)	Vegan (*n* = 48)	Lacto-ovo vegetarian (*n* = 48)	Pesco-vegetarian (*n* = 48)	Semi-vegetarian (*n* = 48)	Regular meat-eater (*n* = 48)	*P*
Energy (kJ)	9560 (9252, 9868)	9550 (8830, 10270)	9690 (8995, 10386)	9185 (8488, 9882)	9836 (9141, 10530.81)	9539 (8840, 10239)	0.753
**Macronutrients (per/day)**							
Protein (%)^a^	16.4% (15.9, 16.9)	15.3% (14.2, 16.4)^*^	14.6% (13.6, 15.7)^*^	16.8% (15.8, 17.9)^*^	15.4% (14.4, 16.5)^*^	20.0% (18.9, 21.1)	**<0.001**
Carbohydrate (%)^a^	40.2% (39.1, 41.3)	43.2% (40.6, 45.7)^*^	41.1% (38.6, 43.6)^*^	38.0% (35.5, 40.5)	42.7% (40.3, 45.2)^*^	36.2% (33.7, 38.7)	**<0.001**
Sugar (g)	87.0 (83.0, 90.9)	76.0 (64.6, 87.3)	93.0 (82.0, 104.0)	83.1 (72.1, 94.1)	91.8 (80.8, 102.7)	91.1 (80.1, 102.2)	0.107
Starch (g)	147.1 (140.4, 153.7)	173.1 (153.5, 192.6)^*^	147.4 (128.4, 166.4)^*^	128.7 (109.7, 147.7)	164.4 (145.5, 183.3)^*^	121.7 (102.7, 140.8)	**<0.001**
Total fat (%)^a^	37.3% (36.3, 38.3)	35.4% (33.0, 37.8)	38.5% (36.2, 40.8)	38.6% (36.3, 40.9)	36.2% (34.0, 38.5)	37.6% (35.3, 39.9)	0.242
Saturated (g)	28.6 (27.3, 30.0)	21.6 (17.9, 25.4)^*^	28.4 (24.8, 32.0)^*^	28.7 (25.1, 32.3)	30.9 (27.4, 34.5)	33.6 (30.0, 37.2)	**<0.001**
Saturated fat (%)^a^	11.1% (10.6, 11.6)	8.7% (7.6, 9.8)^*^	10.8% (9.7, 12.0)^*^	11.4% (10.4, 12.6)	11.7% (10.6, 12.8)	13.0% (11.8, 14.0)	**<0.001**
Trans fats (g)	0.9 (0.8, 1.0)	0.5 (0.3, 0.7)^*^	0.8 (0.7, 1.0)^*^	1.0 (0.8, 1.2)	1.0 (0.8, 1.2)^*^	1.2 (1.1, 1.4)	**<0.001**
Trans fat (%)^a^	0.4% (0.3, 0.4)	0.2% (9.1, 0.3)^*^	0.3% (0.3, 0.4)^*^	0.4% (0.4, 0.5)	0.4% (0.3, 0.44)^*^	0.5% (0.4, 0.5)	**<0.001**
MUFAs (g)	38.7 (37.1, 40.3)	35.9 (31.5, 40.4)	40.1 (35.8, 44.4)	40.5 (36.2, 44.8)	38.3 (34.0, 42.6)	38.9 (34.6, 43.2)	0.242
PUFAs (g)	20.0 (18.9, 21.1)	25.0 (22.1, 27.9)^*^	21.2 (18.3, 24.0)^*^	18.7 (15.9, 21.5)	19.1 (16.3, 21.9)	15.9 (13.1, 18.78)	**<0.001**
Cholesterol (mg)	143.1 (128.9, 157.4)	28.1 (−5.4, 61.5)^*^	93.5 (61.1, 125.8)^*^	161.4 (129.0, 193.8)^*^	158.7 (126.4, 191.0)^*^	274.1 (241.5, 306.6)	**<0.001**
LCn-3PUFA (g)	0.4 (0.4, 0.5)	0.0 (−0.1, 0.2)^*^	0.0 (−0.13, 0.2)^*^	1.0 (0.8, 1.2)	0.4 (0.2, 0.5)^*^	0.8 (0.6, 0.9)	**<0.001**
ALA (g)	2.5 (2.2, 2.8)	3.8 (3.1, 4.5)^*^	2.8 (2.2, 3.5)^*^	2.1 (1.4, 2.8)	2.5 (1.9, 3.2)^*^	1.4 (0.8, 2.1)	**<0.001**
n-6PUFA (g)	17.2 (16.2, 18.3)	21.6 (19.1, 24.0)^*^	18.7 (16.4, 21.1)^*^	15.6 (13.4, 18.0)	16.7 (14.4, 19.1)^*^	13.3 (11, 15.7)	**<0.001**
Dietary fibre (g)	45.3 (43.5, 47.2)	56.9 (51.4, 62.4)^*^	51.5 (46.2, 56.8)^*^	41.8 (36.5, 7.2)^*^	45.1 (39.8, 50.4)^*^	31.4 (26.1, 36.8)	**<0.001**
Alcohol (g)	5.2 (4.0, 6.4)	3.1 (0.3, 6.0)^*^	3.3 (0.6, 6.1)^*^	6.9 (4.1, 9.6)	3.8 (1.1, 6.5)^*^	8.8 (6.0, 11.6)	0.010
**Micronutrients (per/day)**							
Thiamine (mg)	1.5 (1.5, 1.7)	1.9 (1.6, 2.1)	1.6 (1.4, 1.9)	1.3 (1.1, 1.5)^*^	1.6 (1.4, 1.8)	1.6 (1.3, 1.8)	0.039
Riboflavin (mg)	1.7 (1.6, 1.9)	1.5 (1.1, 1.8)^*^	1.7 (1.3, 2.0)^*^	1.7 (1.3, 2.1)^*^	1.8 (1.4, 2.1)^*^	2.3 (1.9, 2.7)	0.031
Niacin (mg)	22.5 (20.7, 24.4)	22.5 (18.1, 26.8)^*^	19.9 (15.7, 24.1)^*^	18.8 (14.6, 3.0)^*^	22.0 (17.9, 26.2)^*^	29.3 (25.1, 33.5)	0.006
Vitamin C (mg)	189.3 (153.7, 225.0)	223.6 (140.0, 307.1)	283.2 (202.3, 364.1)	164.0 (83.0, 244.9)	156.9 (76.4, 237.5)	119.0 (37.8, 200.3)	0.056
Vitamin E (mg)	22.8 (20.5, 25.1)	28.9 (23.5, 34.3)^*^	27.1 (21.8, 32.3)^*^	19.9 (14.7, 25.2)	22.2 (17.0, 27.4)^*^	15.9 (10.6, 21.1)	0.006
Vitamin B_6_ (mg)	1.8 (1.7, 1.9)	2.0 (1.8, 2.2)	1.7 (1.4, 1.9)	1.6 (1.4, 1.8)	1.7 (1.5, 1.9)	1.8 (1.5, 2.0)	0.135
Vitamin B_12_ (µg)	3.2 (2.9, 3.5)	1.4 (0.7, 2.1)^*^	1.8 (1.1, 2.4)^*^	3.8 (3.1, 4.4)	3.3 (2.7, 4.0)	5.7 (5.0, 6.3)	**<0.001**
Total folate (µg)	678.0 ± 266.8	750.3 (675.7, 824.9)^*^	727.9 (655.7, 800.2)^*^	654.3 (582.1, 726.5)	692.3 (620.4, 764.2)^*^	565.1 (492.6, 637.6)	**<0.001**
Vitamin A eq (µg)	1754 (1609, 1899)	1745 (1405, 2084)	1871 (1541, 2199)	1731 (1402, 2059)	1943 (1615, 2270)	1482 (1152, 1812)	0.352
Sodium (mg)	2345 (2066.6, 2622.8)	2136 (1485, 2788)^*^	1916 (1285, 2546)^*^	2166 (1535, 2797)^*^	2263 (1636, 2891)^*^	3242 (2609, 3875)	0.040
Potassium (mg)	4430 (4054, 4804)	4633 (3754, 5512)	5014 (4163, 5865)	4067 (3216, 4919)	4136 (3289, 4984)	4296 (3441, 5151)	0.534
Magnesium (mg)	522.1 (494.4, 549.8)	620.0 (555.1, 684.9)^*^	556.0 (493.2, 618.9)^*^	474.5 (411.7, 537.4)	511.9 (449.3, 574.4)	448.0 (384.9, 511.1)	0.003
Calcium (mg)	1085 (1017, 1152)	955 (796, 1114)	1134 (981, 1288)	1092 (938, 1246)	1086 (933, 1239)	1158 (1004, 1313)	0.436
Phosphorus (mg)	1692 (1603, 1780)	1543 (1336, 1750)	1682 (1482, 1883)	1604 (1403, 1804)	1690 (1492, 1890)	1940 (1739, 2141)	0.075
Iron (mg)	15.0 (14.1, 15.9)	18.6 (16.6, 20.7)^*^	16.1 (14.1, 8.1)^*^	13.4 (11.4, 15.4)	14.5 (12.6, 16.6)	12.4 (10.4, 14.4)	**<0.001**
Zinc (mg)	11.4 (10.3, 12.5)	10.7 (8.2, 13.2)	13.0 (10.5, 15.4)	9.6 (7.1, 12.0)	11.4 (9.0, 13.8)	12.6 (10.1, 15.0)	0.280
Selenium (µg)	75.7 (70.2, 81.2)	64.5 (51.6, 77.3)	65.4 (52.9, 77.9)	82.0 (69.5, 94.5)	81.6 (69.2. 94.0)	85.2 (72.6, 97.7)	0.063
Iodine (µg)	138.8 (128.4, 149.2)	94.7 (70.3, 119.1)^*^	124.8 (101.2, 148.4)^*^	140.1 (116.5, 163.8)^*^	147.6 (124.0, 171.1)^*^	187.0 (163.3, 210.7)	**<0.001**
**Food groups & categories (serves per/day)**^**b**^							
Vegetables	5.9 (5.6, 6.1)	6.4 (5.8, 7.1)^*^	6.1 (5.4, 6.7)^*^	5.9 (5.2, 6.5)^*^	6.0 (5.3, 6.6)^*^	4.9 (4.3, 5.6)	0.033
Starchy	1.0 (0.9, 1.1)	1.0 (0.9, 1.2)	1.2 (1.0, 1.4)	1.0 (0.8, 1.1)	0.9 (0.8, 1.1)	0.9 (0.7, 1.1)	0.136
Non-Starchy	4.8 (4.6, 5.1)	5.4 (4.8, 6.0)^*^	4.9 (4.3, 5.5)^*^	4.9 (4.3, 5.5)^*^	5.0 (4.5, 5.6)^*^	4.0 (3.5, 4.6)	0.040
Grains	2.7 (2.5, 2.9)	2.7 (2.3, 3.1)	2.6 (2.2, 2.9)	2.8 (2.5, 3.2)	2.8 (2.4, 3.2)	2.7 (2.3, 3.0)	0.817
Non-refined	2.1 (2.0, 2.2)	2.2 (1.9, 2.5)	2.0 (1.7, 2.3)	2.3 (1.9, 2.6)	2.0 (1.7, 2.3)	2.0 (1.7, 2.3)	0.567
Refined	0.6 (5.6, 0.7)	0.5 (0.4, 0.7)	0.6 (0.4, 0.7)	0.6 (0.4, 0.7)	0.8 (0.6, 0.9)	0.7 (0.5, 0.8)	0.195
Fruit	3.1 (2.9, 3.3)	3.8 (3.4, 4.3)^*^	3.0 (2.5, 3.4)	2.9 (2.5, 3.4)	3.2 (2.7, 3.7)	2.6 (2.1, 3.0)	0.008
Protein-rich foods^c^	1.9 (1.8, 2.0)	1.5 (1.3, 1.7)^*^	1.3 (1.1, 1.5)^*^	2.0 (1.8, 2.2)^*^	2.0 (1.8, 2.2)^*^	2.7 (2.5, 2.9)	**<0.001**
Meats/seafood^c^	0.6 (0.5, 0.6)	0.0 (−0.1, 0.1)^*^	0.0 (−0.1, 0.1)^*^	0.6 (0.5, 0.7)^*^	0.6 (0.5, 0.8)^*^	1.7 (1.6, 1.9)	**<0.001**
Legumes/nuts	1.1 (1.0, 1.1)	1.5 (1.4, 1.7)^*^	1.1 (0.9, 1.2)^*^	1.1 (0.9, 1.2)^*^	1.0 (0.9, 1.1)^*^	0.6 (0.4, 0.7)	**<0.001**
PBMA^d^	0.2 (0.1, 0.3)	0.4 (0.2, 0.6)^*^	0.3 (0.1, 0.4)^*^	0.2 (0.1, 0.4)^a^	0.1 (−0.2, 0.2)	0.0 (−0.2, 0.2)	0.016
Meat and PBMA	2.1 (2.0, 2.2)	1.9 (1.6, 2.2)^*^	1.6 (1.3, 1.8)^*^	2.2 (2.0, 2.5)^*^	2.0 (1.8, 2.3)^*^	2.7 (2.4, 3.0)	**<0.001**
Dairy	1.6 (1.3, 1.6)	0.0 (−0.3, 0.4)^*^	1.7 (1.3, 2.0)	1.8 (1.5, 2.1)	1.7 (1.4, 2.0)	2.0 (1.7, 2.3)	**<0.001**
PBDA^d^	1.5 (1.3, 1.8)	3.2 (2.7, 3.8)^*^	1.7 (1.2, 2.2)^*^	1.2 (0.7, 1.7)^*^	1.2 (0.7, 1.6)^*^	0.4 (−0.1, 0.9)	**<0.001**
Dairy and PBDA	3.0 (2.7, 3.2)	3.3 (2.7, 3.8)	3.3 (2.8, 3.9)	3.0 (2.4, 3.5)	2.9 (2.3, 3.4)	2.4 (1.8, 2.9)	0.123
Discretionary choices	1.4 (1.3, 1.5)	1.0 (0.7, 1.3)^*^	1.2 (0.9, 1.5)^*^	1.3 (1.0, 1.6)^*^	1.5 (1.2, 1.7)^*^	2.0 (1.7, 2.3)	**<0.001**
Sweetened beverages	0.4 (0.3, 0.4)	0.2 (0.0, 0.4)*	0.3 (0.1, 0.5)^*^	0.3 (0.2, 0.5)^*^	0.4 (0.2, 0.5)^*^	0.6 (0.5, 0.9)	0.006
ARFS	39.3 (38.1, 40.5)	39.8 (37.1, 42.5)^*^	41.1 (38.5, 43.8)^*^	41.5 (38.9, 44.1)^*^	39.0 (36.4, 41.6)^*^	35.1 (32.5, 37.7)	0.005

Data are reported as means (95% CI) and multivariable regression models were used to adjustment for covariates including sex (male, female), age (contentious), BMI ( < 25 kg/m^2^, ≥ 25 kg/m^2^ [32], race (Oceanian, North-West & South-East European, other), total energy intake (%E) and physical activity (MET/week). Dietary intake data derived from an average of two dietitian-administered diet histories and the Australian Eating Survey® food frequency questionnaire. Dietary data does not include mineral/supplement intake. *p ≤ 0.05 marks differences between PBDs and the regular meat diet as the reference group. Values that meet the Bonferroni threshold of 0.0009 (57 different regression models) are bold.

*ARFS* Australian Recommended Food Score, *ALA* α-linolenic acid, *Eq* equivalent, *MUFAs* monounsaturated fatty acids, *PBDA* Plant-based-dairy alternative, *PBMA* plant-based meat alternative, *PUFAs* polyunsaturated fatty acids, *LCn-3PUFA* long chain omega-3 polyunsaturated fatty acids, *n-6PUFA* omega-6 polyunsaturated fatty acids.

^a^Data presented as % contribution of energy.

^b^All food groups and food categories defined by the Australian Guide to Healthy Eating [34].

^c^Protein-rich foods include meats, poultry, seafood, eggs, legumes, and nuts. Assessment of meat exclusion among vegans and LOVs and dairy exclusion among vegans were derived from diet histories.

^d^Plant-based meat and dairy alternatives derived from additional questions included in the food frequency questionnaire.

### Nutritional adequacy of food group intake

**Fig. 1:**
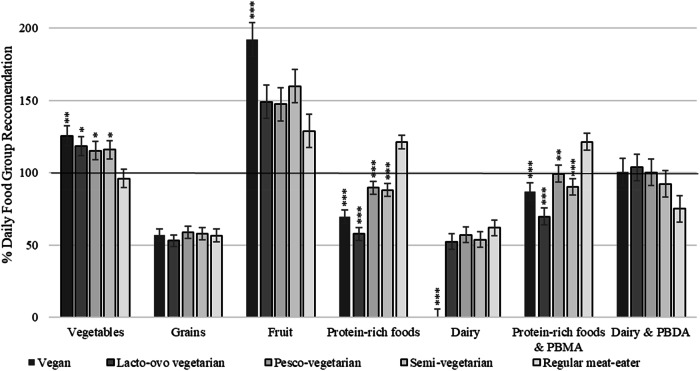
Daily food group intake from plant-based diets compared to the regular meat-eating diet expressed as adjusted mean ± SE, as a percentage (%) of the recommended serves per day. The solid line represents 100% adequacy of food group intake as per the Australian Guide to Healthy Eating (34). Dietary intake data obtained from the Australian Eating Survey® and dietitian administered food frequency questionnaires and does not include mineral/supplement intake. Supplementary Table 3 provides adjusted mean (95% CIs) as a percentage of food group recommendations. PBDA plant-based dairy alternative, PBMA plant-based meat alternative. Protein-rich foods are defined as meats and poultry, seafood, eggs, nuts and seeds, and legumes/beans. Multivariable regression models were used to adjustment for covariates sex, age, BMI, race, total energy intake and physical activity. Asterisks represent comparisons to the regular meat diet as the reference group: *p ≤ 0.05; **p ≤ 0.01; ***p ≤ 0.001.1; ***p ≤ 0.001.

Including plant-based alternatives, all dietary patterns met the recommended intake for fruits and vegetables, however not for grains (Fig. 1). Vegans, LOVs and SVs had inadequate serves of protein-rich foods and SVs and RMEs had inadequate serves of dairy. After combining protein-rich foods and PBMA this elevated levels of adequacy for those following PBDs. The PV group met recommendations and all other PBDs reached >70% adequacy. The combined dairy and PBDA categories also increased levels resulting in adequate intake for vegans, LOVs and PVs, but not SVs or RMEs. Adjusted mean daily intake of core food groups and as a percentage of the AGTHE recommendations can be found in Supplementary Table 2.

### Macronutrient intake

After adjustments, total energy intake was comparable, although intakes of most nutrients were significantly different across groups (Table 2). Protein consumed by RMEs was significantly higher by 3-5% percentage energy intake (EN%) compared to those adhering to a PBD, carbohydrate was lower by 5-7 EN% compared to vegans, LOVs and SVs, and fat (EN%) was comparable. Saturated fat and LCn-3PUFA (nil) intake was significantly lower among vegans and LOVs when compared to RMEs. In contrast, intake of α-linolenic acid (ALA) and omega-6 polyunsaturated fatty acid (n-6PUFA) was significantly higher among vegans compared to RMEs.

### Nutritional adequacy of macronutrients

**Fig. 2:**
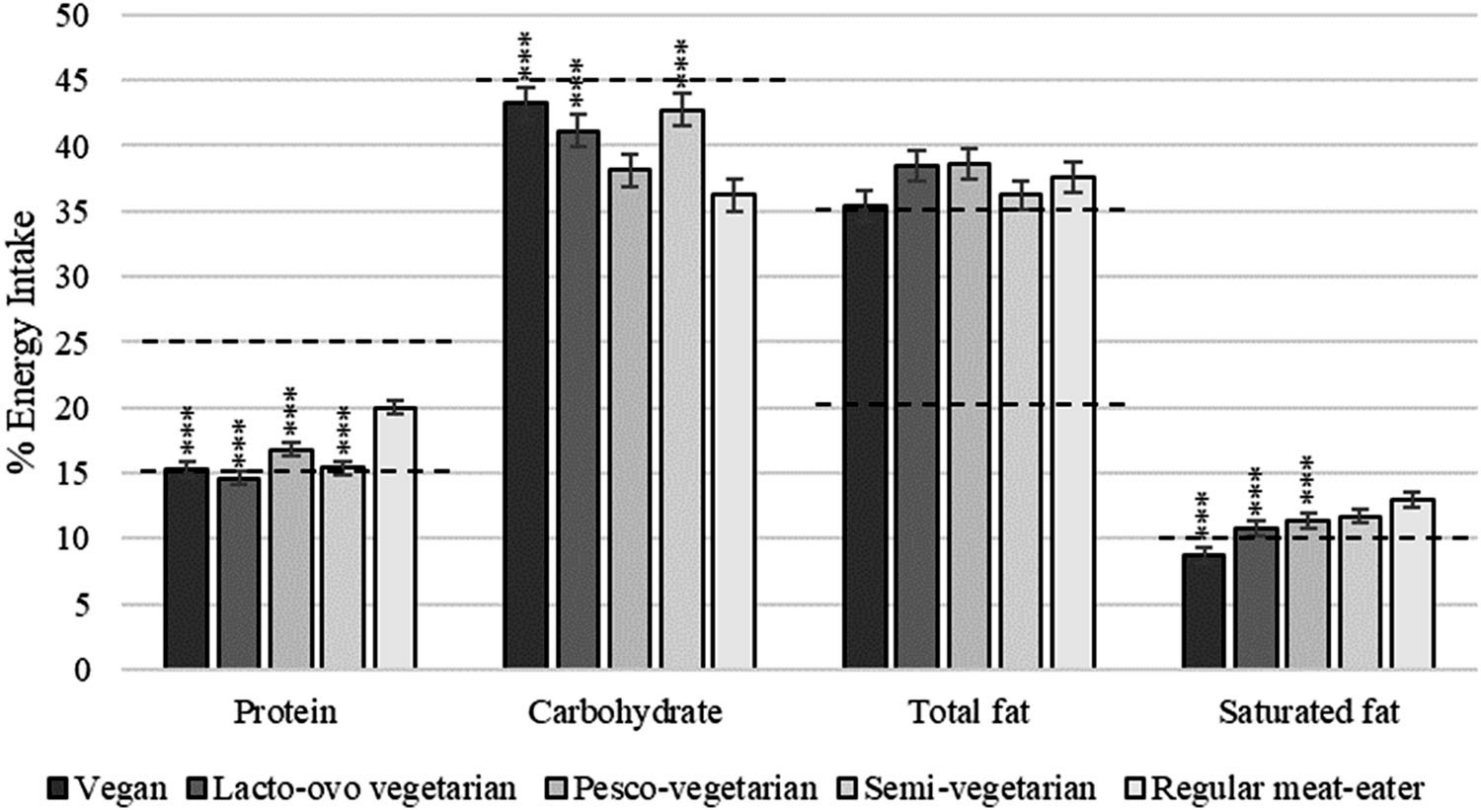
Macronutrient intake from plant-based diets compared to the regular meat-eating diet expressed as adjusted mean ± SE as a percentage of total energy (EN%). Dotted lines represent the upper and lower limits of the Acceptable Macronutrient Ranges (AMDR) as per the Australian dietary guidelines (34). Upper limit for carbohydrates is 65 EN% which is not displayed and intake of saturated fats does not have a lower limit. Dietary intake data obtained from an average of two dietitian-administered diet histories does not include mineral/supplement intake. Multivariable regression models were used to adjustment for covariates sex, age, BMI, race, total energy intake and physical activity. Asterisks represent comparisons to the regular meat diet as the reference group: *p ≤ 0.05; **p ≤ 0.01; ***p ≤ 0.001.

**Fig. 3:**
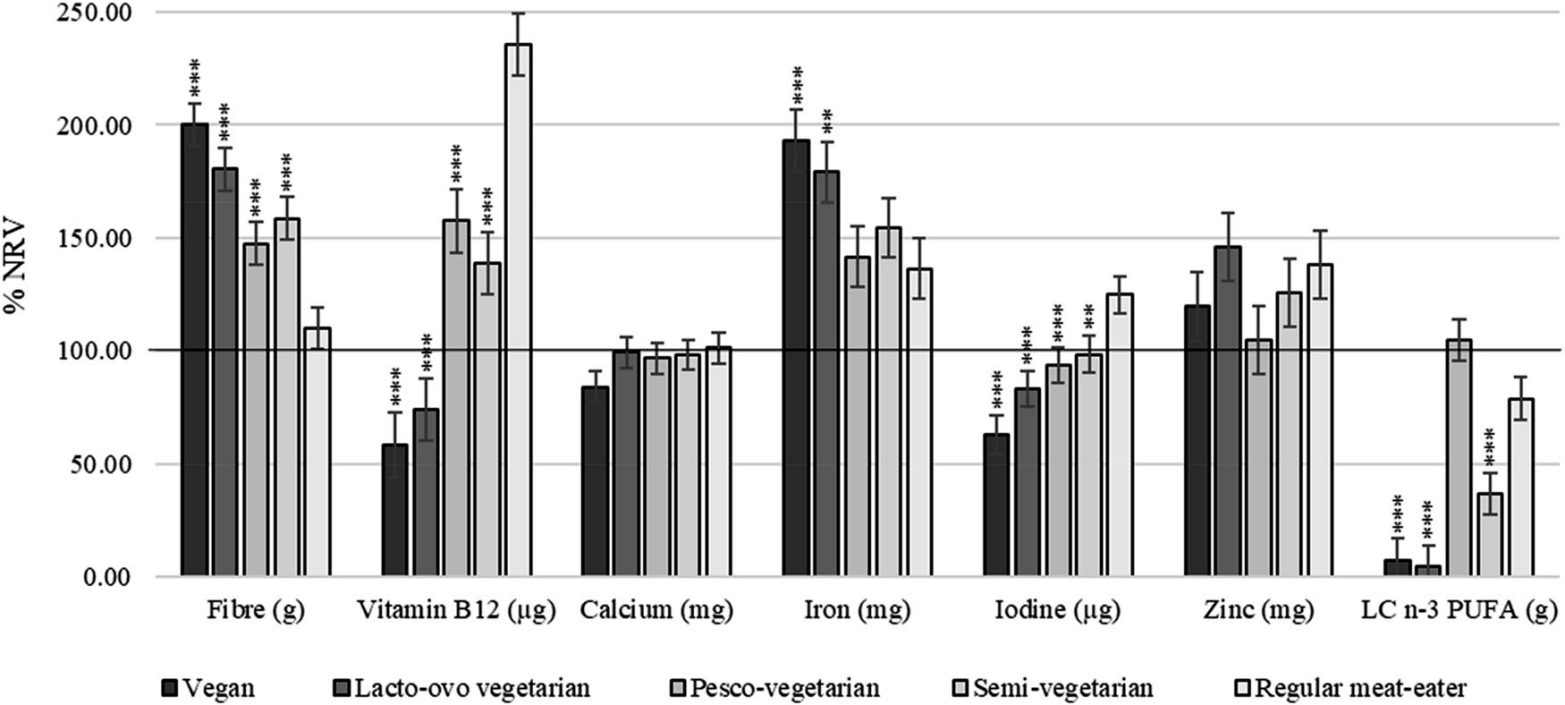
Nutrient intake from plant-based diets compared to the regular meat-eating diet expressed as adjusted mean ± SE, as a percentage (%) of the Nutrient Reference Value (NRV), specific to age and gender recommendations. The solid line represents 100% of the NRV as per the Australian Dietary Guidelines (34). Dietary intake data derived from an average of two dietitian-administered diet histories and does not include mineral/supplement intake. Supplementary Table 2 provides adjusted means (95% CIs) as a percentage of NRVs. LC n-3 PUFA, long chain omega-3 poly unsaturated fatty acids. Multivariable regression models were used to adjustment for covariates sex, age, BMI, race, total energy intake and physical activity. Asterisks represent comparisons to the regular meat diet as the reference group: *p ≤ 0.05; **p ≤ 0.01; ***p ≤ 0.001.

All dietary patterns met the AMDR EN% for protein, exceeded the upper level for fats, and fell below the lower level for carbohydrate (Fig. 2). Those following a vegan dietary pattern were the only group to meet the recommended <10 EN% as saturated fat. PVs consumed adequate intakes of LCn-3PUFA and all other groups fell below recommendations; RMEs, SVs, LOVs and vegans (Fig. 3). All dietary patterns reached adequate dietary fibre intake, although RMEs consumed significantly less than those following a PBD which was almost half the intake of vegans (57g Vs 31g). Adjusted mean daily intakes of all nutrients as a percentage of the NRVs can be found in Supplementary Table 3.

### Micronutrient intake

After adjustments, intake of the following micronutrients were significantly different across dietary patterns; thiamine, riboflavin, niacin, vitamin E, vitamin B_12_, folate, sodium, magnesium, iron, and iodine (Table 2). Compared to RMEs, vegans and LOVs consumed higher amounts vitamin E, folate, magnesium, and iron, whereas SVs and PVs had intermediate intakes. In contrast, those following a PBD consumed lower amounts of riboflavin, niacin, sodium, iodine, and the addition of vitamin B_12_ in vegans and LOVs only, in comparison to RMEs. Vegans and LOVs consumed fourfold less vitamin B_12_ and around half the amount of iodine compared to RMEs. Intake of nutrients that were not significantly different across dietary patterns include vitamin C, vitamin A, vitamin B_6,_ vitamin A, potassium, calcium, phosphorus, zinc, and selenium.

### Nutritional adequacy of micronutrients

Nutritional inadequacies were more likely to occur in vegans and LOVs than RMEs (Fig. 3). After adjustments, vegans and LOVs had inadequate intake of vitamin B_12_, LCn-3PUFA, iodine, and in addition, calcium in vegans, PVs in iodine, and SVs and RMEs in LCn-3PUFA. All groups met the recommended intake for iron and vegans consumed two times the NRV. Intake of all other nutrients were adequate for all dietary patterns.

### Effects of covariates on nutrient intakes

After adjusting for potential confounding variables including sex, age, BMI, total energy intake and physical activity, the effect of dietary patterns on nutrient intakes did not lose significance. Adjusted *P*-values have been reported in tables.

## Discussion

This cross-sectional study found that compared to RMEs, individuals following vegan, and LOV dietary patterns had a more favourable nutrient composition including lower intakes of saturated fat, trans fat, sodium and cholesterol, higher intakes of dietary fibre, vitamin E, folate, magnesium, iron, ALA, and n-6PUFA, whereas PVs and SVs were intermediate. Those adhering to a PBD consumed significantly more daily serves of vegetables, fruit (vegans only), legumes/nuts and less discretionary choices and sugar sweetened beverages in comparison to the regular-meat diet. Evaluating nutrient intakes against the dietary guideline recommendations revealed all dietary patterns met adequate intake for protein (EN%), exceeded for fat (EN%) were below for carbohydrate (EN%), had adequate serves of fruit and vegetables, but not grains. Moreover, vegans, LOVs, and SVs had inadequate serves of protein-rich foods, and SVs and RMEs had inadequate serves of dairy. Regarding micronutrients, vegans and LOVs had inadequate intake of LCn-3PUFA, vitamin B_12_, iodine, and in addition, calcium in vegans, PVs in iodine, and SVs and RMEs in LCn-3PUFA.

Studies that investigate nutritional intake of PBDs are generally scarce, especially within the Australian context, therefore our current understanding is largely from secondary analyses of overseas prospective cohort studies including the ‘EPIC Oxford Study’ (*n* = 65,429) [23] and the ‘Adventist Health Study-2 (AHS-2)’ (*n* = 71,751) [25], and moderate sized cross-sectional studies [44, 45]. Protein-rich foods are predominantly of animal origin including meat, poultry and fish, although relatively high levels of protein can also be found from plant-based sources such as legumes and nuts [46]. Our findings suggest that those following meat-free or low meat PBDs consume adequate amounts of protein (15–17 EN% vs the AMDR of 15–25 EN%), consistent with a similar cross-sectional cohort study which found that vegans, LOVs and SVs consume 13–14 EN% from protein. These findings also align with results from the larger ‘EPIC oxford’ cohort [23, 44] which states LOVs and vegans typically consume sufficient (or borderline) amounts of protein to meet daily requirements, provided a variety of protein-rich foods are consumed and energy requirements are met [45, 47]. Moreover, the ‘Nurses Health Study’ (*n* = 48,762) prospective cohort found that plant-based protein, are associated with higher odds of healthy ageing and lower risk of chronic illness [48].

Vegans and LOVs are known to consume higher amounts of carbohydrates compared to dietary patterns inclusive of animal products [45, 49], likely driven by the higher intake of carbohydrate-rich plant foods namely legumes/pulses, fruit and grains [49]. This was reflected in the current study as vegans and LOVs were observed to have a higher carbohydrate intake (EN%), although none of the dietary patterns reached the recommended carbohydrate AMDR (40 EN% vs 45–65 EN%). This was akin to the larger cohort studies including the ‘EPIC Oxford Study’ and ‘AHS-2’ which detailed vegans to consume the highest amount of carbohydrates (54–61 EN%) in comparison to LOVs (49–57 EN%) and RMEs (46–57 EN%) [23, 25, 44]. In the present study intake of dietary fibre among vegans was over twice the NRV (25–30 g/day), although all dietary patterns reached adequate intake. These results are supported by previous cross-sectional and prospective cohort studies which demonstrated vegans to consume 20–70% above the recommended 30 g/day, however, RMEs have previously been reported to not meet recommendations [25, 44, 45]. The most recent 2011–2012 National Nutrition and Physical Activity Survey reported 7 in 10 adults did not meet the adequate dietary fibre intake, further establishing general levels of inadequacy among those following a RME diet [50]. Above adequate dietary fibre among RMEs within this sample could be attributed towards the ‘healthy user effect’ which explains health conscious individuals who become involved in research may have healthier lifestyle behaviours and dietary patterns [51].

All groups marginally exceeding AMDR for dietary fat intake (37% vs 20–35 EN%). This agrees with previous observational studies which have reported vegans to consume 26–28 EN%, LOVs 30–33 EN% and RMEs 32–35 EN% from dietary fats [23, 25, 44, 45]. Moreover, such studies describe vegans, LOVs, PVs, and SVs to have the lowest intake of saturated fat, trans fats and cholesterol and highest intakes of PUFAs compared to RMEs, reflective of the current study [23, 25, 44, 45]. Vegans achieved <10 EN% (recommended level) from saturated fat and as expected, had a notably lower intake of dietary cholesterol, seven times less than RMEs, similar to the a cross-sectional study in healthy adults which demonstrated a ten times difference [44]. Dietary fat profiles may be partially explained by protein sources, for example plant-based protein such as legumes/pulses, nuts, seeds are predominantly being rich in unsaturated fats, whilst consuming minimal/nil animal-based foods which are inherently a rich source of saturated and trans fats [52]. Our findings demonstrated that all dietary patterns except for PVs did not meet the recommended intake for LCn-3PUFA. Vegans and LOVs consumed <10% of the recommended NRV, therefore could be considered most at risk of LCn-3PUFA inadequacy, and essential nutrient. These findings are also in accordance with moderate size cross-sectional studies from Germany which reported inadequate intake amongst individuals adhering to both PBDs and RME diets [44, 45]. Noteworthy, this is not necessarily a characteristic specific only to individuals following PBDs, since the 2011–2012 National Nutrition and Physical Activity Survey (the most recent at the time) found 80% of the Australian population did not meet recommendations for LCn-3PUFA [53]. LCn-3PUFA intake continues to be observed at critically low level of consumption for both PBDs and meat-eating diets within the current study population but represents a broader issue among the Australian adult population.

Despite the healthful nature of PBDs, there is potential for nutrient inadequacies to occur given the restriction of key food groups such as dairy, eggs and animal meats. Of most concern is iron, zinc, iodine, calcium, and vitamin B_12_ which are essential for human health including growth and development, immune health, production of hormones, bone health and neurological functioning [20, 54]. Calcium intake was inadequate in vegans by 16%. Previous studies report inconclusive outcomes whereby similar cross-sectional studies reported 25–40% of vegans to not meet recommendations for calcium [44, 45]. On the other hand, alike the current study, the ‘AHS-2’ cohort detailed most PBDs (SV, PV, and LOV) reached recommendations with only 7% of vegans to fall short [25]. The inclusion of vitamin and mineral-fortified food products in the current study may explain the higher intake levels of calcium as well as vitamin B_12_ when compared to previous studies. Low dietary intake of calcium among the vegans in this study may be explained by the fact that over half of the Australian population aged ≥2 years have inadequate intake of calcium intake from foods, with females reporting a higher prevalence of inadequacy than male [55]. Moreover, while calcium is found in plant foods such as leafy greens [56], milk and milk-based products are the riches sources of dietary calcium among Australians [57]. Given the omission of dairy products as inherent to the vegan dietary pattern, and the majority of individuals following a vegan diet in this study were females, it is no surprise that dietary calcium was observed to be low in this group [56].

Vitamin B_12_ intake was observed in a descending order parallel to restrictiveness of animal products since vitamin B_12_ is usually derived from animal-based foods [58]. It is for this reason that the Australian dietary guidelines recommend strict vegans to supplement with vitamin B_12_ [42]. Those adhering to a PBD also rely on B_12_-enriched food products [58], such as plant-based alternatives and nutritional yeast which were frequently consumed by individuals within this cohort and are commonly spread in the current food supply globally [59]. The NRV for vitamin B_12_ was met by meat-eating groups (RME, SV, PV), although intakes of vegans and LOVs were 42% and 26% below recommendation, respectively. Other previously mentioned cross-sectional and prospective studies mirror results and found LOVs, SVs and PVs to meet recommendations, however, vegans fell below [23, 25, 44, 45]. Of interest, around 50% of the total sample of vegans in this study were taking vitamin B_12_ supplements which would contribute to improved nutritional adequacy.

Iron intake has been shown to be higher in PBDs compared to RME diets as 80–90% of dietary iron intake is non-haem [25]. Non-haem iron is derived from plant-sources such as beans, legumes, nuts and leafy greens, whereas haem iron is derived from animal sources such as meats and poultry [60]. The amount of iron derived from non-haem food sources is often much higher than that of haem food sources [25]. In this study we observed vegans and LOVs to consume 1–1.5 more daily serves of vegetables 0.5–1 additional serves of legumes and nuts which explains the higher overall iron intake among PBDs. The food structure and bioavailability play a key role when analysing iron adequacy. Haem iron from animal-based food is readily absorbed (20–30% rate), while the absorption of non-haem iron from plant-based foods is less available (1–10% rate) [61]. It is for this reason that the Institute of Medicine states vegetarians and vegans require 1.8 times higher dietary iron compared to non-vegetarians [61]. Those following PBDs (vegan, LOV, PV, and SV) in this sample reached this additional requirement which is in accordance with previous literature [44, 62]. When comparing this samples iron intake to the Australian dietary guidelines all groups met the recommended intake for iron and vegans consumed two times the NRV. At current the Australian dietary guidelines do not recommend any additional iron intake for those following PBDs [42]. Bioavailability can also strongly depend on dietary factors and an individual’s current iron status [61]. Inhibitors such as phytates, oxalates, fibre, calcium and polyphenols can bind and interfere with absorption of non-heam iron, while vitamin C rich food such a citrus fruits and vegetables can simultaneously enhance absorption [61]. High fibre dietary patterns such as PBDs may lead to an increase consumption of inhibitors which compromise absorption rates [61]. Despite vitamin C intake being comparable across groups, vegans and LOVs tended to have higher intakes compared to RMEs which may play a role in increasing bioavailability of non-haem iron. This may be amplified by use of vitamin C supplements which was reported at a comparable level (13–27% being users). Therefore, it is pivotal to use biochemical measures to ascertain the true iron status of individuals adhering to a PBD.

Zinc and iodine are two trace minerals that are typically consumed in lower amounts when following vegan and LOV dietary patterns and their bioavailability is also affected by the presence of inhibitors such as phytic acid [44]. Intake of zinc was adequate and comparable across groups, aligning with a previous cross-sectional study and large observational studies [23, 25, 45]. Noteworthy, in one study LOVs, vegans and PVs males were slightly below recommendations [25] and LOVs males in another study [45]. Additional analyses not shown in results tables of the current study also show all males following PBDs fell just below (5–20%) recommended intakes. The Australian dietary guidelines recommend vegetarians to consume 50% more zinc as majority of plant-based protein-rich foods (also rich in zinc) are absorbed at a lower rate than animal proteins, and this combined with higher consumption of legumes and grains may exceed the phytate zinc ratio (15:1) [63]. None of the individuals adhering to a PBD met the additional 50% requirement for zinc.

Iodine consumption was markedly different between vegans and RMEs equating to a 70% difference and most PBDs fell below recommendations by 37% for vegans, 17% for LOVs and 7% for PVs. Literature on dietary iodine intake is scarce, however, one similar cross sectional cohort study reported 60% of vegans and LOVs did not reach requirements [44]. Like other nutrients, iodine content in plant-based foods are much lower compared to that of animal origin [64]. Therefore, urinary iodine concentrations are more commonly used to assess iodine levels as it provides an objective and more accurate reading. Previous studies examining urinary iodine have found vegans to be moderately iodine deficient and LOVs and PVs mildly deficient [65], however further research is warranted.

This study is the first to measure daily food group intake of Australians following various PBDs in accordance with the national recommendations as per the AGTHE. Those following a PBD did not reach the recommended serves per day for ‘protein-rich foods’ and dairy (without inclusion of plant-based alternatives). However, when including intake of PBDAs, intake of dairy was adequate across all PBDs, except SVs (7% below). This could explain why those adhering to PBDs (excluding vegans) met the recommended AMDR for protein and calcium. Moreover, the combined category of PBMA with ‘protein-rich foods’ evaluate adequacy of individuals following PBDs by 7–10%, enabling PVs to reach recommended serves and all other PBDs to reach >70% adequacy. This points out the necessity for dietary collection tools to encompass the abundance of plant-based alternative food products available within society to better evaluate daily food group intake amongst those following PBDs. It was found that individuals adhering to a PBD consumed greater quantities of food groups such as fruit, vegetables and grains compared to RMEs and lower intakes of sugar sweetened beverages and discretionary choices. This may suggest PBDs obtain energy intake predominantly from wholefood food groups. Moreover, PBDs consumed significantly more daily serves of legumes, nuts and seeds compared to other RMEs, which may be a key driver for the higher levels of PUFA. A similar cross-sectional pilot study in athletic adults corroborated results reporting that vegans and LOVs had a higher vegetable, fruit, wholegrain, and legume intake compared to RME [45]. In addition, the intake of legumes among vegans was 18 times more than RMEs, higher than the current study which was two times greater. More research surrounding PBDs and food group intake using validated FFQs inclusive of plant-based alternatives is warranted to substantiate trends observed in the current study [66].

Strengths of this study include its novel study design that specifically recruited individuals habitually following a variety of PBDs and RME diets from the Australian population. It utilised a variety of validated tools including the AES FFQ^®^ and experienced APDs to collect quantitative dietary intake data from interviewer-administered diet histories, thus providing a greater level of accuracy. In the absence of a validated population-based FFQ specific for PBDs, this study design enabled a better canvas of the current plant-based food and beverage (including fortified items) supply and thus resulted in a truer representation of dietary intake in the current climate. Moreover, it was the first Australian-based study to our knowledge to compare nutrient and daily food group intake of PBDs to the NRVs/AGTHE recommendations.

Several limitations must be addressed. First, vitamin and mineral supplementation use were not included in the final analysis due to irregular usage, variations in brand and bioavailability and possibility of skewing the data. Second, vegans were significantly younger than the other diet groups which may have affected usual eating habits and nutrient intakes, although age was adjusted for in all analyses. Third, most data collected was self-reported, however, the data collection tools implemented in this study have been validated in the Australian population [58], and interviewer-administered research methods by APDs were used to strengthen collection of subjective measures. Fourth, the dietary analysis software used was unable to quantify data on animal versus plant-derived iron and vitamin D intake. Analysis of nutritional status indicated by biochemical measures were outside the scope of this study, however, the researchers acknowledge that this would verify the findings. More information on plasma blood lipids has been published elsewhere [67]. Fifth, this study was a secondary analysis on a modest sample size from the same geographical region with low reported prevalence of chronic disease so results should be interpreted with caution and may not be generalised to all Australians. Lastly FFQs can be prone to some measurement error including under or over reporting due to memory, inability to capture all foods consumed, day-to-day variation and food preparation methods [68]. Conversely, validated FFQs are the most accurate form of measurement of the intake of food items relating to a food category over a long period of time, therefore such measures were used to measure qualitative dietary intake [35]. Diet histories were used to collect further detail on dietary intake as well as measure quantitative dietary data. Diet histories have been considered more reliable than food weighed records [35, 41], however can be prone to some limitations such as recall bias, interviewer bias and errors in portion size estimations [69]. Efforts were made to mitigate these limitations such as administration by a trained APDs, taking an average of two diet histories to inform analyses, and completing each diet history with a dietary checklist to ensure no food category or eating occasion were missed. This study could provide guidance for the design and conduct, including its consistent method of categorising and tailored dietary collection of PBDs, for forthcoming larger observational and/or interventional studies.

In summary, findings from this cross-sectional cohort which purposefully sampled a variety of Australians habitually following PBDs, revealed vegan and LOV dietary patterns offer some advantages in nutrient composition including lower saturated fats, trans fats, cholesterol and sodium and higher intakes of dietary fibre, n-6PUFA, ALA, vitamin E, iron, folate, and magnesium, whereas PVs and SVs were intermediate in comparison to RMEs. Regarding levels of nutritional adequacy all dietary patterns met adequate intake for protein, exceeded for fat, and were below for carbohydrate (EN%), had adequate serves of fruit and vegetables, but not grains. Vegans, LOVs, and SVs had inadequate serves of protein-rich foods, and SVs and RMEs had inadequate serves of dairy. Rates of nutritional inadequacies varied across groups, vegan and LOVs were deficient in vitamin B_12_, LCn-3PUFA, iodine, and in addition calcium among vegans, PVs in iodine, and RMEs and SVs in LCn-3PUFA. Our study contributes to current population-based evidence on the nutritional intake of PBDs in middle-aged adults in Australia which is consistent with findings from similar overseas cohorts [25, 44]. Findings may aid the development of national dietary strategies and guidelines to achieve nutritional adequacy for those following plant-based eating patterns, in congruence with the updated dietary guidelines around the globe. Nutrients of concern among PBDs and RMEs as well as suitability of supplementation should be carefully considered by individuals alongside tailored nutrition interventions with their healthcare professionals when adjusting dietary intakes to meet nutritional needs. Moreover, nutrients found to be in deficit should be considered when designing plant-based alternatives. Larger primary population-based longitudinal studies investigating nutrient intakes and objective biomarkers are warranted to substantiate nutritional status of various PBDs.

## Supplementary information

Supplemental Material

## Data Availability

The datasets used and/or analysed during the current study are available from the corresponding author on reasonable request.
