# Supplementary material for: Dietary intakes and nutritional adequacy of Australians consuming plant-based diets compared to a regular meat-eating diet
Source: Eur J Clin Nutr. 2025 Apr 18;79(9):876–87. doi: 10.1038/s41430-025-01621-1 (PMC12422967; doi:10.1038/s41430-025-01621-1)
Supplement: Supplementary file 1 — Supplemental Material [file 41430_2025_1621_MOESM1_ESM.docx]

**Table S1.** Unadjusted means for quantitative and qualitative dietary intake of macronutrients, micronutrients, and food groups across dietary patterns.

| **Nutrients/Food groups (per/day)** | **Total sample (n=240)** | **Vegan  (n=48)** | **Lacto-ovo vegetarian (n=48)** | **Pesco-vegetarian (n=48)** | **Semi-vegetarian (n=48)** | **Regular meat-eater (n=48)** | ***P*** |
| --- | --- | --- | --- | --- | --- | --- | --- |
| Energy (kJ) | 9560 ± 2650 | 9957 ± 2712 | 9709 ± 3325 | 9013 ± 2284 | 9631 ± 2501 | 9491 ± 2304 | 0.506 |
| **Macronutrients** |  |  |  |  |  |  |  |
| Protein (%)^1,2^ | 16.4% ± 4.1 | 15.4% ± 3.6^a,b^ | 14.8% ± 4.2^a^ | 16.8% ± 3.2^b^ | 15.4% ± 2.8^a,b^ | 19.8% ± 4.5^c^ | <0.001 |
| Protein (g)^1^ | 92.1 ± 38.0 | 90.8 ± 34.3^a,b^ | 86.1 ± 62.6^a^ | 87.2 ± 20.9^a^ | 86.3 ± 23.6^a^ | 110.0 ± 28.9^b^ | 0.007 |
| Carbohydrate (%)^1,2^ | 40.2% ± 9.4 | 43.9% ± 8.4^a^ | 41.3% ± 8.9^a,b^ | 37.9% ± 8.9^b,c^ | 42.3% ± 8.7^a,b^ | 35.8% ± 9.9^c^ | <0.001 |
| Sugar (g) | 87.0 ± 39.1 | 77.3 ± 26.4 | 91.8 ± 46.4 | 82.6 ± 32.0 | 90.5 ± 29.1 | 92.7 ± 53.1 | 0.221 |
| Starch (g) | 147.1 ± 76.5 | 184.7 ± 86.9^a^ | 152.1 ± 78.7^a,b^ | 124.9 ± 54.8^b^ | 115.1 ± 62.5^a,b^ | 115.5 ± 62.5^b^ | <0.001 |
| Total fat (%)^1,2^ | 37.3% ± 8.1 | 35.2% ± 8.4 | 38.2% ± 8.4 | 38.6% ± 7.8 | 36.5% ± 7.1 | 33.2% ± 14.6 | 0.227 |
| Saturated (g)^1^ | 28.6 ± 13.2 | 23.3 ± 11.2^a^ | 28.4 ± 13.9^a,b^ | 27.8 ± 13.3^a,b^ | 30.4 ± 11.1^a,b^ | 33.2 ± 14.6^b^ | 0.003 |
| Saturated fat (%)^2^ | 11.1% ± 0.4 | 8.7% ± 4.1^a^ | 10.8% ± 3.1^a^ | 11.4% ± 1.1^b^ | 11.7% ± 3.1^b^ | 13.0% ± 3.6^b^ | <0.001 |
| Trans fats (g)^1^ | 1.0 ± 0.6 | 0.5 ± 0.7^a^ | 0.8 ± 0.5^a^ | 1.0 ± 0.6^b^ | 1.0 ± 0.6^b^ | 1.2 ± 0.6^b^ | <0.001 |
| Trans fat (%)^2^ | 0.4% ± 0.2 | 0.2% ± 0.3^a^ | 0.3% 0.2^a^ | 0.4% ± 0.3^b^ | 0.4% ± 0.2^b^ | 0.5% ± 0.2^b^ | <0.001 |
| MUFAs (g)^1^ | 38.7 ± 15.1 | 37.0 ± 15.6 | 40.1 ± 15.8 | 39.9 ± 16.1 | 37.7 ± 14.6 | 39.1 ± 13.9 | 0.835 |
| PUFAs (g)^1^ | 20.1 ± 10.3 | 25.8 ± 13.6^a^ | 21.2 ± 10.0^a,b^ | 18.3 ± 7.4^b^ | 18.8 ± 9.8^b^ | 15.8 ± 6.8^b^ | <0.001 |
| Cholesterol (mg)^1^ | 143.1 ± 136.5 | 33.6 ± 92.1^a^ | 98.2 ± 120.8^a,c^ | 157.9 ± 108.2^b^ | 155.7 ± 110.6^b,c^ | 270.2 ± 128.6^d^ | <0.001 |
| Dietary fibre (g)^1^ | 45.3 ± 20.8 | 58.0 ± 18.8^a^ | 50.4 ± 29.7^a^ | 41.5 ± 13.6^b,c^ | 44.3 ± 16.9^b^ | 32.5 ± 11.0^c^ | <0.001 |
| Alcohol (g)^1^ | 5.2 ± 10.1 | 1.8 ± 4.3^a^ | 3.5 ± 7.1^a^ | 7.2 ± 12.0^a,b^ | 4.1 ± 7.8^a,b^ | 9.4 ± 14.4^b^ | 0.001 |
| **Micronutrients** |  |  |  |  |  |  |  |
| Thiamine (mg) | 1.6 ± 0.9 | 1.9 ± 0.9^a^ | 1.7 ± 1.1^a,b^ | 1.3 ± 0.4^b^ | 1.5 ± .07^a,b^ | 1.5 ± 0.9^a,b^ | 0.003 |
| Riboflavin (mg) | 1.8 ± 1.3 | 1.6 ± 0.8 | 1.6 ± 1.6 | 1.7 ± 0.7 | 1.7 ± 0.9 | 2.3 ± 2.0 | 0.061 |
| Niacin (mg) | 22.5 ± 15.0 | 23.2 ± 15.3^a,b^ | 19.9 ± 14.8^a^ | 18.5 ± 6.3^a^ | 21.5 ± 8.1^a^ | 29.5 ± 22.7^b^ | 0.003 |
| Vitamin C (mg) | 189.3 ± 281.1 | 218.3 ± 122.6 | 273.3 ± 595.0 | 165.8 ± 86.8 | 159.0 ± 85.8 | 130.2 ± 67.5 | 0.096 |
| Vitamin E (mg) | 22.8 ± 18.8 | 29.5 ± 28.8^a^ | 26.8 ± 24.1^a^ | 19.6 ± 8.2^a,b^ | 21.9 ± 12.3^a,b^ | 16.3 ± 6.9^b^ | 0.003 |
| Vitamin B_6_ (mg) | 1.8 ± 0.8 | 2.0 ± 1.2 | 1.6 ± 0.8 | 1.6 ± 0.5 | 1.7 ± 0.6 | 1.8 ± 0.6 | 0.081 |
| Vitamin B_12_ (µg) | 3.2 ± 2.7 | 1.6 ± 1.2^a^ | 1.8 ± 1.3^a^ | 3.7 ± 2.1^a,b^ | 3.3 ± 2.2^a,b^ | 5.6 ± 3.6^c^ | <0.001 |
| Total folate (µg) | 678.0 ± 266.8 | 778.5 ± 270.0^a^ | 724.1 ± 337.9^a^ | 646.4 ± 251.1^a,b^ | 678.1 ± 242.7^a,b^ | 562.9 ± 160.8^b^ | 0.001 |
| Vitamin A eq (µg) | 1754 ± 1145 | 1720 ± 1201 | 1835 ± 1223 | 1725 ± 950 | 1945 ± 1347 | 1546 ± 990 | 0.521 |
| Sodium (mg) | 2345 ± 2255 | 2341 ± 1118 | 2084 ± 836 | 2060 ± 784 | 2189 ± 941 | 3050 ± 4661 | 0.167 |
| Potassium (mg) | 4430 ± 2944 | 4645 ± 1881 | 4924 ± 5500 | 4052 ± 964 | 4116 ± 1330 | 4410 ± 2653 | 0.571 |
| Magnesium (mg) | 522.1 ± 233.4 | 640.1 ± 275.6^a^ | 553.9 ± 296.7^a,b^ | 466.3 ± 136.4^b^ | 502.6 ± 166.9^b^ | 447.5 ± 205.0^b^ | <0.001 |
| Calcium (mg) | 1085 ± 548 | 1026 ± 531 | 1124 ± 594 | 1063 ± 383 | 1068 ± 433 | 1144 ± 740 | 0.831 |
| Phosphorus (mg) | 1692 ± 723 | 1633 ± 589 | 1667 ± 8856 | 15712 ± 381 | 16567 ± 437 | 1930 ± 1055 | 0.131 |
| Iron (mg) | 15.0 ± 7.4 | 19.4 ± 6.4^a^ | 16.2 ± 12.0^a,b^ | 13.1 ± 4.0^b^ | 14.2 ± 4.4^b^ | 12.3 ± 5.1^b^ | <0.001 |
| Zinc (mg) | 11.4 ± 8.4 | 11.3 ± 4.2 | 12.7 ± 17.4 | 9.3 ± 2.5 | 11.2 ± 3.4 | 12.6 ± 3.9 | 0.269 |
| Selenium (µg) | 75.7 ± 43.9 | 67.2 ± 57.1 | 66.4 ± 45.7 | 80.4 ± 32.7 | 79.6 ± 48.0 | 84.9 ± 27.9 | 0.114 |
| Iodine (µg) | 138.8 ± 88.9 | 106.1 ± 56.7^a^ | 124.2 ± 64.4^a^ | 137.2 ± 73.8^a,b^ | 144.0 ± 94.0^a,b^ | 182.7 ± 123.6^b^ | <0.001 |
| LCn-3PUFA (g) | 0.4 ± 0.7 | 0.0 ± 0.0^a^ | 0.0 ± 0.0^a,b^ | 1.0 ± 0.8^c^ | 0.4 ± 0.6^d^ | 0.8 ± 0.8^c^ | <0.001 |
| ALA (g) | 2.5 ± 2.4 | 3.7 ± 3.4^a^ | 2.8 ± 2.1^a,b^ | 2.1 ± 1.3^b^ | 2.5 ± 2.9^a,b^ | 1.5 ± 0.9^b^ | <0.001 |
| n-6PUFA (g) | 17.2 ± 8.8 | 22.3 ± 10.7^a^ | 18.9 ± 8.8^a^ | 15.3 ± 6.8^b^ | 16.3 ± 8.1^b^ | 13.3 ± 6.1^b^ | <0.001 |
| **Food groups & categories^3^** | |  |  |  |  |  |  |
| Vegetables^1^ | 5.9 ± 2.4 | 6.3 ± 2.5 | 6.2 ± 2.6 | 5.9 ± 2.1 | 5.9 ± 2.1 | 5.0 ± 2.6 | 0.081 |
| Starchy | 1.0 ± 0.6 | 0.9 ± 0.7 | 1.2 ± 0.8 | 1.0 ± 0.6 | 0.9 ± 0.5 | 0.9 ± 0.5 | 0.129 |
| Non-Starchy | 4.8 ± 2.1 | 5.3 ± 2.0 | 5.0 ± 2.2 | 4.9 ± 1.9 | 5.0 ± 1.8 | 4.1 ± 2.5 | 0.058 |
| Grains^1^ | 2.7 ± 1.3 | 2.7 ± 1.1 | 2.6 ± 1.3 | 2.8 ± 1.5 | 2.7 ± 1.3 | 2.6 ± 1.5 | 0.907 |
| Non-refined | 2.1 ± 1.1 | 1.1 ± 1.0 | 2.0 ± 1.1 | 2.3 ± 1.4 | 2.0 ± 1.0 | 2.0 ± 1.2 | 0.568 |
| Refined | 0.6 ± 0.5 | 0.5 ± 0.5 | 0.6 ± 0.5 | 0.6 ± 0.4 | 0.8 ± 0.6 | 0.7 ± 0.5 | 0.103 |
| Fruit^1^ | 3.1 ± 1.7 | 3.8 ± 2.1^a^ | 3.0 ± 1.3^a,b^ | 3.0 ± 1.6^a,b^ | 3.1 ± 1.5^a,b^ | 2.7 ± 1.6^b^ | 0.015 |
| Protein-rich foods^1,4^ | 1.9 ± 0.9 | 1.5 ± 0.7^a^ | 1.3 ± 0.5^a^ | 2.0 ± 0.6^b^ | 2.0 ± 1.0^a,b^ | 2.7 ± 0.7^c^ | <0.001 |
| Meats/seafood^1,4^ | 0.6 ± 0.8 | 0.0 ± 0.0^a^ | 0.0 ± 0.0^a^ | 0.6 ± 0.4^b^ | 0.6 ± 0.7^a,d^ | 1.7 ± 0.6^c^ | <0.001 |
| Legumes/nuts^1^ | 1.1 ± 0.6 | 1.5 ± 0.7^a^ | 1.1 ± 0.5^b^ | 1.1 ± 0.5^b^ | 1.0 ± 0.5^b^ | 0.6 ± 0.5^c^ | <0.001 |
| PBMA^4^ | 0.2 ± 0.6 | 0.4 ± 0.8^a^ | 0.3 ± 0.8^a,b^ | 0.2 ± 0.7^a,b^ | 0.1 ± 0.1^b^ | 0.0 ± 1.1^b^ | 0.008 |
| Meat and PBMA | 2.1 ± 1.0 | 1.9 ± 0.9^a,b^ | 1.6 ± 0.9^a^ | 2.2 ± 1.0^b,c^ | 2.0 ± 1.0^a,b^ | 2.7 ± 0.7^c^ | <0.001 |
| Dairy^1^ | 1.6 ± 1.3 | 0.0 ± 0.0^a^ | 1.6 ± 1.5^b^ | 1.8 ± 1.0^b^ | 1.7 ± 1.3^b^ | 2.0 ± 1.4^b^ | <0.001 |
| PBDA^5^ | 1.5 ± 1.9 | 3.2 ± 2.0^a^ | 1.7 ± 1.9^b^ | 1.2 ± 1.9^b,c^ | 1.2 ± 1.6^b,c^ | 0.4 ± 1.1^c^ | <0.001 |
| Dairy and PBDA | 3.0 ± 1.9 | 3.2 ± 2.0 | 3.3 ± 2.2 | 3.1 ± 1.9 | 2.9 ± 1.7 | 2.4 ± 1.7 | 0.171 |
| Discretionary choices^1^ | 1.7 ± 1.2 | 1.3 ± 1.0 ^a^ | 1.6 ± 1.1 ^a^ | 1.6 ± 1.1 ^a^ | 1.8 ± 1.2^a,b^ | 2.3 ± 1.5^b^ | 0.002 |
| Sweetened beverages | 0.4 ± 0.6 | 0.3 ± 0.4^a^ | 0.3 ± 0.4^a^ | 0.3 ± 0.5^a^ | 0.3 ± 0.7^a,b^ | 0.6 ± 0.8^b^ | 0.016 |
| ARFS | 39.3 ± 9.5 | 40.0 ± 9.1^a,b^ | 40.8 ± 8.1^a^ | 41.3 ± 11.3^a^ | 38.6 ± 8.5^a,b^ | 35.9 ± 9.5^b^ | 0.006 |

ARFS, Australian Recommended Food Score; ALA, α-linolenic acid; eq, equivalent; MUFAs, monounsaturated fatty acids; PBDA, Plant-based-dairy alternative; PBMA, plant-based meat alternative; PUFAs, polyunsaturated fatty acids; LCn-3PUFA, long chain omega-3 polyunsaturated fatty acids; n-6PUFA, omega-6 polyunsaturated fatty acids. Data are reported as absolute means ± SD and *p*-values reported from ANOVA. Dietary intake data derived from an average of two dietitian-administered diet histories and the Australian Eating Survey® food frequency questionnaire.

^1^Unadjusted macronutrients data has been published elsewhere (28)

^2^Data presented as % contribution of energy.

^3^All food groups and food categories defined by the Australian Guide to healthy eating (34).

^4^Protein-rich foods include meats, poultry, seafood, eggs, legumes, and nuts. Assessment of meat exclusion among vegans and LOVs and dairy exclusion among vegans were derived from diet histories.
^5^Plant-based meat and dairy alternatives derived from dietitian administered food frequency questionnaire.

^a,b,c,d^Values within a row without a common superscript letters are significantly different (*P* < 0.05).

**Table S2.** Food group intake expressed as adjusted means of the number of serves consumed daily and as a percentage (%) of recommendations daily serves per the Australian Guide to Healthy Eating (AGTHE) across dietary patterns.

| **Food groups** | **Total sample (n=240)** | **Vegan  (n=43)** | **Lacto-ovo vegetarian (n=55)** | **Pesco-vegetarian (n=41)** | **Semi-vegetarian (n=46)** | **Regular meat-eater (n=55)** |
| --- | --- | --- | --- | --- | --- | --- |
| Vegetables | 114.2 (108.6, 119.9) | 125.6 (112.4, 138.9) | 118.4 (105.6, 131.2) | 115.3 (102.4, 128.1) | 115.8 (103.0, 128.6) | 96.0 (83.1, 108.9) |
| Grains^1^ | 56.6 (52.9,  60.3) | 56.9 (48.3,  65.5) | 52.9 (44.5,  61.2) | 58.6 (50.3,  67.0) | 57.9 (49.6,  66.2) | 56.6 (48.2,  64.9) |
| Fruit | 155.5 (145.4, 165.5) | 192.1 (168.6, 215.7) | 149.1 (126.3, 171.9) | 147.3 (124.5, 170.1) | 159.8 (137.1, 182.5) | 128.9 (106.0, 151.8) |
| Protein-rich foods^2^ | 85.2 (81.2,  89.1) | 69.6 (60.3,  78.8) | 57.7 (48.7,  66.6) | 89.5 (80.5,  98.4) | 87.9 (79.0.  96.8) | 121.2 (112.2, 130.2) |
| Meat and PBMA^3^ | 93.5 (88.4,  98.6) | 86.9 (75.0,  98.8) | 69.7 (58.2,  81.2) | 99.3 (87.8, 110.8) | 90.2 (78.7, 101.7) | 121.4 (109.9, 133.0) |
| Dairy | 44.4 (39.7,  49.0) | 0 (-14.4,  7.5) | 52.4 (41.8,  63.0) | 57.1 (46.5,  67.8) | 53.7 (43.1,  64.3) | 62.0 (51.3,  72.6) |
| Dairy and PBDA^3^ | 94.3 (86.4, 102.3) | 100.4 (81.8, 119.0) | 103.6 (85.6, 85.6) | 100.2 (82.2, 118.2) | 92.3 (74.4, 110.3) | 75.0 (57.0,  93.1) |

PBDA, Plant-based-dairy alternative; PBMA, Plant-based meat alternative; AGTHE, Australian Guide to Healthy Eating. Qualitative dietary intake data is presented as an average from the Australian Eating Survey® Food Frequency Questionnaire. Model 1 results are derived from a multivariable regression model adjusted for sex (male, female), age (contentious), BMI (<25kg/m2, ≥ 25 kg/m2 (31), race (Oceanian, North-west & South- European, other), total energy intake (%E) and physical activity (MET/week) are reported as means (95% CI).

^1^All food groups and food categories recommended serves defined by the AGHTE (34).

^2^Protein-rich foods’ include meats/poultry/seafood/eggs/legumes/nuts. Assessment of meat exclusion among vegans and LOVs and dairy exclusion among vegans were derived from diet histories.
^3^Plant-based meat and dairy alternatives derived from additional questions included in the FFQ. Recommendations for ‘protein-rich foods’ and dairy were as per the AGTHE (34).

**Table S3.** Adjusted micronutrient intake expressed as a percentage of the nutrient reference value (NRV%) appropriate for age and gender as per the Australian Dietary Guidelines.

| **Micronutrients (per/day)^1^** | **Total sample (n=240)** | **Vegan  (n=48)** | **Lacto-ovo vegetarian (n=48)** | **Pesco-vegetarian (n=48)** | **Semi-vegetarian (n=48)** | **Regular meat-eater (n=48)** |
| --- | --- | --- | --- | --- | --- | --- |
| Thiamine (mg) | 141.7 (132.4, 150.9) | 163.6 (141.8, 185.3) | 149.1 (128.0, 170.1) | 115.2 (94.1, 136.2) | 141.6 (120.7, 162.6) | 138.8 (117.7, 159.9) |
| Riboflavin (mg) | 155.3 (140.5, 170.0) | 127.5 (92.9, 162.0) | 144.9 (111.4, 178.3) | 147.6 (114.1, 181.0) | 153.5 (120.2, 186.8) | 202.9 (169.3, 236.5) |
| Niacin (mg) | 155.0 (142.1, 167.8) | 153.3 (123.1, 183.5) | 137.1 (107.9, 166.4) | 129.6 (100.4, 158.8) | 151.5 (122.4, 180.6) | 203.3 (173.9, 232.6) |
| Vitamin C (mg) | 420.8 (341.6, 500.1) | 496.7 (311.1, 682.4) | 629.8 (450.1, 809.6) | 364.4 (184.6, 544.2) | 348.7 (169.7, 527.7) | 264.5 (83.9, 45.0) |
| Vitamin E (mg) | 301.2 (269.7, 332.8) | 381.7 (307.8, 455.6) | 360.1 (288.6, 431.6) | 260.2 (188.7, 331.8) | 292.9 (221.6, 364.1) | 211.3 (139.4, 283.1) |
| Vitamin B6 (mg) | 121.0 (113.8, 128.2) | 140.0 (123.2, 156.8) | 112.5 (96.3, 128.8) | 113.0 (96.7, 129.2) | 117.6 (101.4, 133.8) | 121.8 (105.5, 138.2) |
| Vitamin B12 (µg) | 132.8 (120.7, 144.8) | 58.1 (29.8, 86.5) | 73.9 (46.5, 101.3) | 157.5 (130.1, 184.9) | 138.6 (111.3, 165.9 | 235.6 (208.1, 263.2) |
| Total folate (µg) | 169.5 (161.6, 177.5) | 187.4 (168.8, 206.1) | 182.3 (164.2, 200.3) | 163.6 (145.5, 181.7) | 173.1 (155.1, 191.10) | 141.3 (123.1, 159.4) |
| Vitamin A eq (µg)^2^ | 237.9 (218.3, 257.5) | 235.8 (189.9, 281.7) | 254.7 (210.2, 299.1) | 236.1 (191.6, 280.6) | 262.2 (217.9, 306.5) | 201.0 (156.3, 245.6) |
| Sodium (mg) | 254.2 (224.0, 284.4) | 231.5 (160.8, 302.3) | 205.7 (137.2, 274.2) | 235.3 (166.8, 303.8) | 246.0 (177.8, 314.2) | 352.4 (283.6, 421.2) |
| Potassium (mg) | 148.0 (134.8, 161.2) | 154.0 (123.1, 184.8) | 169.4 (139.5, 199.3) | 135.3 (105.4, 165.2) | 136.9 (107.2, 166.7) | 144.4 (114.4, 174.4) |
| Magnesium (mg) | 152.6 (144.5, 160.8) | 180.5 (161.4, 199.7) | 163.4 (144.9, 181.9) | 138.0 (119.4, 156.5) | 149.4 (130.9, 167.9) | 131.9 (113.3, 150.5) |
| Calcium (mg) | 95.9 (90.0, 101.7) | 83.9 (70.2, 97.7) | 99.3 (86.0, 112.7) | 96.7 (83.3, 110.0) | 98.2 (84.9, 111.4) | 101.1 (87.7, 114.5) |
| Phosphorus (mg) | 169.0 (160.2, 177.8) | 153.7 (133.0, 174.4) | 167.9 (147.9, 188.0) | 160.3 (140.3, 180.4) | 169.0 (149.1, 189.0) | 194.0 (173.9, 214.2) |
| Iron (mg) | 160.9 (149.3, 172.5) | 192.9 (165.7, 220.0) | 179.1 (152.8, 205.3) | 141.6 (115.3, 167.8) | 154.6 (128.5, 180.8) | 136.3 (110.0, 162.7) |
| Zinc (mg) | 126.8 (113.7, 139.9) | 119.5 (88.8, 150.2) | 146.1 (116.4, 175.8) | 104.8 (75.0, 134.5) | 125.5 (95.9, 155.1) | 138.2 (108.3, 168.1) |
| Selenium (µg) | 121.2 (112.2, 130.2) | 103.5 (82.5, 124.6) | 104.1 (83.7, 124.5) | 131.7 (111.4, 152.1) | 130.1 (109.8, 150.4) | 136.7 (116.3, 157.2) |
| Iodine (µg) | 92.5 (85.5, 99.4) | 62.8 (46.6, 79.1) | 83.1 (67.4, 98.8) | 93.4 (77.7, 109.2) | 98.4 (82.7, 114.0) | 124.7 (108.9, 140.4) |
| LCn-3PUFA (g) | 46.3 (38.2, 54.4) | 7.1 (-11.8, 26.0) | 4.3 (-14.1, 22.6) | 104.6 (86.3, 123.0) | 36.7 (18.4, 54.9) | 78.8 (60.3, 97.2) |
| ALA (g) | 286.4 (251.4, 321.4) | 436.6 (354.5, 518.6) | 314.0 (234.6, 393.4) | 230.7 (151.3, 310.2) | 287.4 (208.3, 366.5) | 163.3 (83.5, 243.1) |
| n-6PUFA (g) | 193.8 (182.0, 205.6) | 245.0 (217.3, 272.7) | 212.2 (185.4, 239.0)^a^ | 172.2 (145.4, 199.0) | 189.1 (162.4, 215.8) | 150.6 (123.7, 177.5) |
| Fiber (g) | 159.3 (151.1, 167.4) | 200.0 (180.9, 219.1) | 180.5 (162.0, 199.0) | 147.4 (128.9, 165.9) | 158.7 (140.3, 177.1) | 109.9 (91.4, 128.5) |

ALA, alpha-linolenic acid; eq, equivalent; LC n-3 PUFA, long chain omega-3 poly unsaturated fatty acids; nutrient reference value, NRV; N-6PUFA, omega-6 poly unsaturated fatty acids. Quantitative dietary intake data is presented as an average of two Dietitian-administered diet histories. Crude data are reported as means ± SD. Model 1 results are derived from a multivariable regression model adjusted for sex (male, female), age (contentious), BMI (<25kg/m2, ≥ 25 kg/m2 (31), race (Oceanian, North-west & South- European, other), total energy intake (%E) and physical activity (MET/week) are reported as means (95% CI). Percentage NRVs based of adjusted data.

^1^ All NRVs defined by the AGHTE. When the Recommended Daily Intake was not available the Estimated Average Requirements, Adequate Intake, or Upper Level was used to calculate nutrient adequacy for adults. All nutrient intakes were matched to age and sex NRV recommendations. The higher value of the upper limit was used for sodium (920mg) (42).

^2^NRV for retinol equivalents (µg) were used to compare adequacy of vitamin A equivalents (µg).
